# Supplementary figures and images for: Trophoblast glycoprotein is required for efficient synaptic vesicle exocytosis from retinal rod bipolar cells
Source: Front Cell Neurosci. 2023 Nov 30;17:1306006. doi: 10.3389/fncel.2023.1306006 (PMC10720453; doi:10.3389/fncel.2023.1306006)

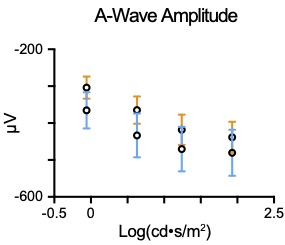

Supplement: Supplementary Figure 1 — TPBG-KO does not alter the scotopic ERG a-wave. Quantification of a-wave amplitudes recorded after light stimuli [−0.06 to 1.93 log(cd⋅s/m2)]. A-waves are measured from baseline to the a-wave trough. Data is presented as the mean ± SEM. [file Image_1.jpeg]

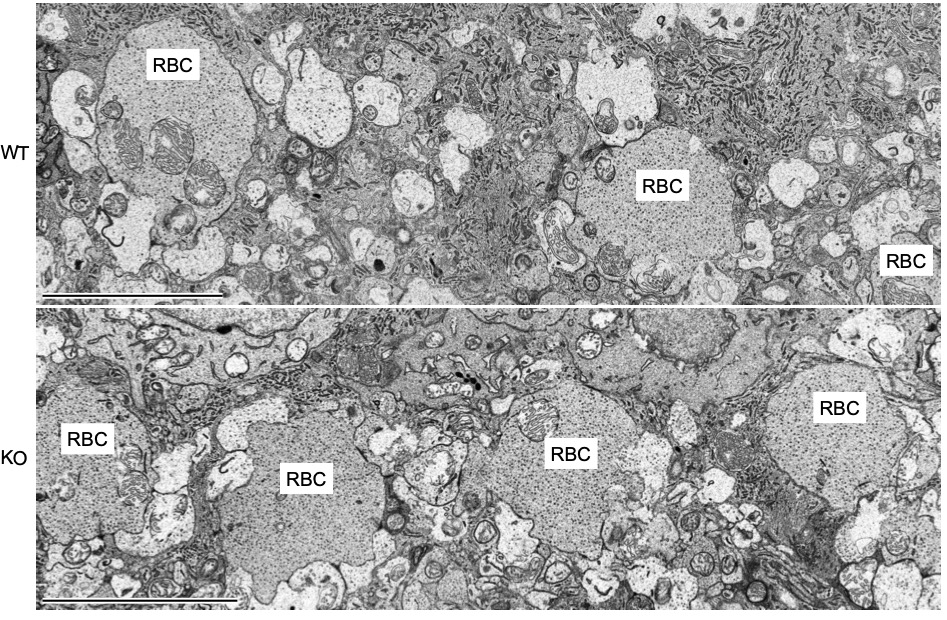

Supplement: Supplementary Figure 2 — FIB-SEM imaging of RBC axon terminals. Focused ion beam scanning electron microscopy (FIB-SEM) was used to image RBC axon terminals in the distal IPL and examine synaptic ribbon location and morphology. Zoomed-out examples of WT and TPBG-KO (KO) RBC axon terminals in the IPL. The scale bar is 5 μm. [file Image_2.jpeg]

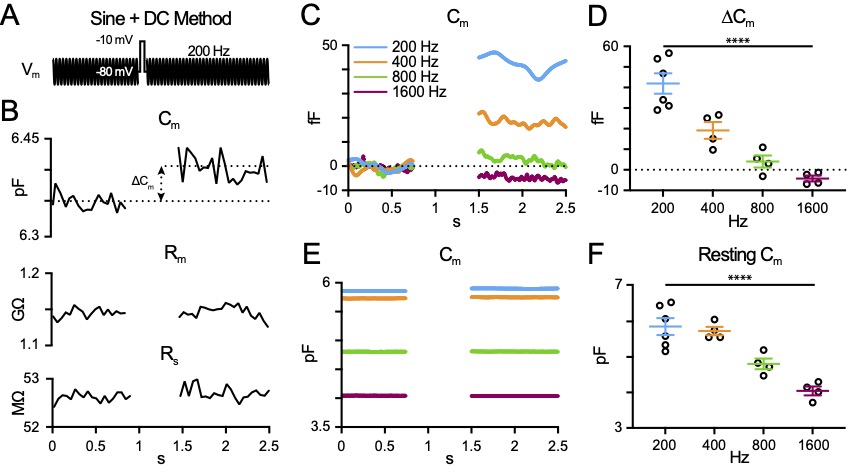

Supplement: Supplementary Figure 3 — Measuring membrane capacitance in RBCs. (A) Whole-cell voltage-clamp capacitance recordings were made using the “sine + DC” method. A sinusoidal voltage command (200 Hz at 30 mV trough-to-peak) was superimposed on the holding potential of −80 mV. Membrane capacitance (Cm), membrane resistance (Rm), and series resistance (Rs) were recorded before and after a depolarizing pulse from −80 mV to −10 mV for 100 ms. (B) A sample recording from a WT RBC using the sine + DC method as described. The change in membrane capacitance (ΔCm) was measured as the difference between the Cm before and after the depolarizing pulse. Changes to Rm (middle) and Rs (bottom) were small and do not correlate with changes in Cm. (C) Average baseline-subtracted Cm recordings using 200 Hz (blue; n = 6), 400 Hz (orange; n = 4), 800 Hz (green; n = 4), or 1,600 Hz (magenta; n = 4) sinusoidal voltage commands in WT RBCs. Data for the 200 Hz condition was reused from WT in Figure 4. Traces with higher frequencies were resampled to 200 Hz for ease of comparisons. (D) Quantification of ΔCm. (E) Average Cm recordings and (F) quantification of resting Cm across frequencies. Resting Cm was measured before the depolarizing pulse. Open circles in bar graphs represent single cells and bars represent the mean ± SEM. Statistical significance was determined using Brown-Forsythe ANOVA tests; ****p < 0.0001. [file Image_3.jpeg]
